# Real-time segmentation and phenotypic analysis of rice seeds using YOLOv11-LA and RiceLCNN

**DOI:** 10.3389/fpls.2025.1673143

**Published:** 2025-12-08

**Authors:** Dejia Zhang, Shaozhong Song, Jia Liu, Weiwei Xu, Nurdila Xiayidan

**Affiliations:** 1 School of Artificial Intelligence, Changchun University of Science and Technology, Changchun, China; 2 Zhongshan Institute of Changchun University of Science and Technology, Zhongshan, China; 3 School of Data Science and Artificial Intelligence, Jilin Engineering Normal University, Changchun, China; 4 College of Electrical and Information Engineering, Jilin Engineering Normal University, Changchun, China; 5 College of Life Sciences and Agri-forestry, Southwest University of Science and Technology, Mianyang, China

**Keywords:** YOLOv11-LA, RiceLCNN, rice seeds, object detection, classification

## Abstract

**Introduction:**

The real-time, accurate detection and classification of rice seeds are crucial for improving agricultural productivity, ensuring grain quality, and promoting smart agriculture. Although significant progress has been made using deep learning, particularly convolutional neural networks (CNNs) and attention-based models, earlier methods such as threshold segmentation and single-grain classification faced challenges related to computational efficiency and latency, especially in high-density seed agglutination scenarios. This study addresses these limitations by proposing an integrated intelligent analysis model that combines object detection, real-time tracking, precise classification, and high-accuracy phenotypic measurement.

**Methods:**

The proposed model utilizes the lightweight YOLOv11-LA for real-time grain segmentation, which builds upon the YOLOv11 architecture. YOLOv11-LA incorporates several enhancements over YOLOv11, including separable convolutions, CBAM (Convolutional Block Attention Module) attention mechanisms, and module pruning strategies. These modifications not only improve detection accuracy but also significantly reduce the number of parameters by 63.2% and decrease computational complexity by 51.6%. For classification, the model employs a custom-designed, lightweight RiceLCNN classifier. Additionally, the DeepSORT algorithm is employed for real-time multi-object tracking, and sub-pixel edge detection along with dynamic scale calibration mechanisms are applied for precise phenotypic feature measurement.

**Results:**

Compared to YOLOv11, the YOLOv11-LA model increases the mAP@0.5:0.95 score by 1.9%, showcasing its superior detection performance while maintaining lower computational overhead. The RiceLCNN classifier achieved classification accuracies of 89.78% on private datasets and 96.32% on public benchmark datasets. The system demonstrated high accuracy in measuring phenotypic features such as seed size and roundness, with measurement errors kept within 0.1 millimeters. The DeepSORT algorithm effectively managed multi-object tracking, reducing duplicate identifications and frame loss in real-time.

**Discussion:**

Experimental validation confirmed that the YOLOv11-LA model outperforms the original YOLOv11 in terms of both detection speed and accuracy, while also maintaining low computational complexity. The integration of the YOLOv11-LA, RiceLCNN, and DeepSORT algorithms, combined with advanced measurement techniques, underscores the model's potential for industrial applications, particularly in enhancing smart agricultural practices.

## 1 Introduction

Rice (*Oryza sativa* L.), as a globally significant staple crop, plays a crucial role in maintaining food security and agricultural economics through its yield and quality. Achieving efficient and precise rice seed detection and classification not only enables stringent quality control at the source and supports the breeding and promotion of superior varieties, but also provides critical technological support for the transformation of agricultural production toward intelligent and precision farming (Karunasena et al., 2020).

Compared to other crops, rice seeds are tightly enclosed by hard inner and outer glumes (husk), and their phenotypic characteristics pose a series of unique challenges for phenotypic analysis. First, rice grains are small and elongated, often appearing in high-density, inter-contacted distributions during high-throughput imaging, significantly increasing the difficulty of target segmentation (Aukkapinyo et al., 2019; Sun et al., 2014; Ning et al., 2023); Second, visual differences between varieties are often subtle, primarily manifesting in traits such as grain length-to-width ratio, end contour, and surface texture; Environmental and cultivation management factors (e.g., day-night temperature differences, water and fertilizer conditions) further amplify intraspecific variation and reduce interspecific distinguishability by influencing quality traits like chalkiness (Zhou et al., 2018; Huo et al., 2023); Additionally, variations in husk surface gloss and color, along with localized high-reflectance and low-contrast regions, complicate feature extraction and threshold segmentation (Yan et al., 2018; Yang et al., 2023).

Traditional rice variety classification methods—manual phenotypic identification, chemical or biological analysis—remain valuable for accuracy and interpretability. However, they are generally destructive time-consuming, and costly. Consequently, recent research has shifted toward image-based deep learning approaches (Cui et al., 2019; Li et al., 2022). In the typical scenario of “dense small objects in close contact,” object detection has become the mainstream technical approach: Mask-based methods excel in contour delineation but are prone to over-segmentation or under-segmentation in densely packed rice seed scenarios, and are not conducive to high-throughput initial screening. In contrast, one-stage methods (YOLO series) maintain accuracy while offering real-time performance. They enable rapid initial screening of rice seeds in detection-priority pipelines, followed by subsequent fine segmentation and measurement, aligning better with high-throughput workflows from field to laboratory. Previously, models like YOLOv5 have demonstrated strong performance in rice seed detection tasks (Phan et al., 2023); recent enhancements—including more efficient backbones and decoupled detector heads, improved small object training and sampling strategies, and end-to-end/low-latency inference—further boost their applicability in dense scenarios. YOLOv9 introduced the Generalized Efficient Layer Aggregation Network (GELAN) and programmable gradient information to enhance information utilization in lightweight models (Wang et al., 2024b); YOLOv10 achieved NMS-free training and significantly reduced latency through strategies like consistent dual allocation, pushing the performance boundaries of small object detection and real-time deployment (Wang et al., 2024a). Building upon this evolutionary trajectory, YOLOv11 comprises three major modules: the backbone network, the neck network, and the head network. The backbone handles multi-scale feature extraction, while the neck aggregates features through multi-layer convolutions and attention mechanisms before passing them to the head, which generates the final predictions. Performance improvements stem primarily from innovative module designs: replacing traditional C2f with C3k2 enhances computational efficiency, and integrating Spatial Pyramid Pooling Fast (SPPF) with Cross-Stage Partial with Spatial Attention (C2PSA) further strengthens feature representation. Furthermore, YOLOv11 undergoes systematic optimization in model size and computational efficiency, demonstrating exceptional performance in multi-scale small object detection and proving particularly well-suited for edge computing scenarios (Rasheed and Zarkoosh, 2025). Accordingly, we build the segmentation frontend upon the lightweight YOLOv11-LA network to balance speed and accuracy while ensuring seamless integration with subsequent fine-grained classification modules.

For seed classification, RiceSeedNet employs a visual Transformer architecture supplemented by traditional image processing for RGB-based rice variety identification (Rajalakshmi et al., 2024); RiceNet focuses on color and shape feature extraction, demonstrating strong generalization capabilities across varying grain sizes and contours (Mohi Ud Din et al., 2024). These studies demonstrate that convolutional models based on standard image information can effectively characterize rice seed phenotype. However, when confronted with high-density, intra-class-diverse real-world workflows, classification networks alone struggle to meet the efficiency and accuracy demands of end-to-end processing. Therefore, this study adopts an integrated “detection front-end – fine-grained classification back-end” approach to balance high throughput and high accuracy in complex scenarios.

Experiments employed a single-factor randomized block design. The same rice variety was planted across nine different treatment combinations (straw incorporation, enzyme application, and various combinations of organic and chemical fertilizers) on plots demonstrating fertilizer efficacy, ensuring consistent cultivation practices. Post-harvest, rice seed samples were collected from each treatment plot and photographed against a standard black background (each image containing 200 grains) to analyze phenotypic variations induced by different fertilizer treatments. Methodologically, we employed an optimized lightweight YOLOv11-LA frontend for automated detection/segmentation of high-density grains, coupled with a lightweight RiceLCNN for phenotypic feature extraction and classification of individual seeds, forming a scalable two-stage segmentation-classification pipeline. The main contributions of this study are as follows: (1) Proposed a lightweight, structurally optimized YOLOv11-LA localization front-end tailored for dense, overlapping, low-contrast rice seed images, enabling real-time inference and consistently improving small object recall; (2) Designs the lightweight and efficient RiceLCNN classification network, enhancing its ability to recognize subtle phenotypic variations in rice seeds; (3) Constructs a rice seed classification dataset under standardized imaging conditions, providing data support for subsequent phenotypic analysis and management strategy evaluation; (4) Establishes a scalable “two-stage segmentation-classification” pipeline, facilitating real-time deployment and online iterative refinement.

## 2 Related work

In rice seed identification experiments, while individual grain imaging can reduce errors, it incurs extremely high labor and time costs; In practice, a more common approach involves capturing multiple grains in a single frame, followed by segmentation. Existing research, often prioritizing classification, typically employs the classical Otsu global threshold (Otsu, 1979) for segmentation. This method achieves unsupervised segmentation by maximizing inter-class variance and has introduced several improvements for rice seed scenarios to enhance robustness under complex lighting and background conditions (Qingge et al., 2020). However, under conditions of low contrast, noise interference, and grain adhesion, Otsu and its variants remain reliant on local adaptation and post-processing, with limited effectiveness in separating adhered objects. Therefore, we turn to detection-driven real-time segmentation: The YOLO series has demonstrated its detection and localization capabilities in agricultural imagery, such as size estimation based on YOLOv7 (Pawłowski et al., 2024) and grain measurement leveraging rotation awareness with YOLOv8 (Zhao et al., 2023). Within this framework, we adopt an optimized and lightweight YOLOv11-LA as the frontend. It inherits the CSP backbone and FPN+PAN feature fusion from YOLOv11n to enhance small object representation. Combined with pruning and quantization, it achieves a balance between accuracy and speed, enabling real-time instance-level segmentation and screening of densely packed, contacting grains.

Based on the instance regions output by this frontend, we employ the lightweight RiceLCNN for single-grain classification and phenotypic feature extraction. Early rice seed classification studies predominantly utilized machine learning algorithms such as Logistic Regression (LR), Linear Discriminant Analysis (LDA), k-Nearest Neighbors (k-NN), and Support Vector Machines (SVM). These methods perform well under conditions of thorough preprocessing and explicit feature extraction. For instance, Kiratiratanapruk et al. (2020) achieved 90.61% accuracy using SVM classification on RGB images. Kurade et al. (2023) combined geometric features with a Random Forest (RF) classifier to achieve a 77% classification rate. Phan et al. (2024) further proposed a hybrid model integrating deep feature extraction with traditional classifiers, enhancing model stability and generalization capabilities. As CNNs and their variants gain widespread adoption for rice variety image classification, Jin et al. (2024) enhanced image quality and training sample selection efficiency by integrating the EDSR network with the Kennard-Stone algorithm. Sharma et al. (2024) introduced transfer learning to rice variety recognition. Comparing architectures like ResNet50, Xception, and InceptionV3, they found ResNet50 performed optimally on large-scale datasets, achieving 80.5% accuracy. These studies demonstrate deep learning’s distinct advantages in feature extraction and phenotypic recognition. To further enhance model inference capabilities, some research attempts to incorporate multi-model fusion into rice variety classification systems. For instance, Rathnayake et al. (2023) fused GBDT with ANFIS to construct a nonlinear classifier, enabling intelligent assessment of rice seed aging. Although such models demonstrate strong classification capabilities, their complex structures and high computational costs limit practical deployment in edge computing and field environments. In contrast, Mohi Ud Din et al. (2024) proposed RiceNet, centered on a lightweight convolutional architecture. Tested on five common rice varieties, it achieved 94% accuracy, outperforming InceptionV3 (84%) and InceptionResNetV2 (81.33%). RiceNet’s compatibility with high accuracy and low computational complexity makes it suitable for rapid deployment and industrialization under resource-constrained conditions. Nevertheless, this approach primarily focuses on static image recognition, and future work should extend it to multi-task scenarios (such as counting and multi-attribute analysis) to enhance practicality.

## 3 Materials and methods

**Figure 1:**
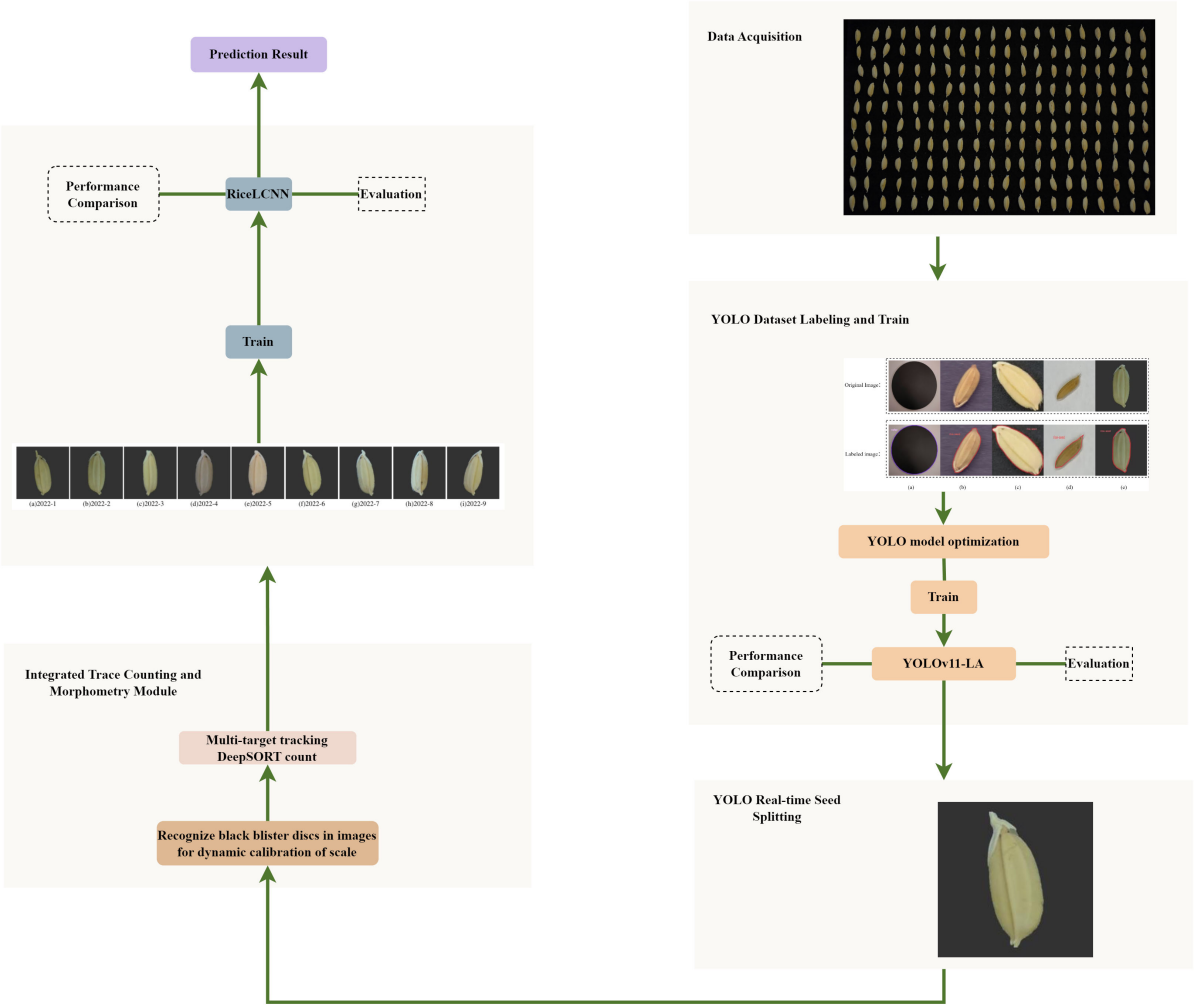
Overall flow chart for rice seed identification.

This study proposes a two-stage segmentation–classification framework for rice seed recognition and phenotypic analysis, consisting of two core modules: (1) the real-time grain segmenter YOLOv11-LA, designed to perform rapid detection and instance segmentation in dense contact scenarios; and (2) the rice seed classifier RiceLCNN, responsible for phenotypic discrimination and feature extraction from individual grain images. As illustrated in **Figure 1**, the overall workflow comprises dataset construction (collection, annotation, and quality control), model design and optimization (architecture refinement, pruning–quantization, and training strategies), tracking-based counting (instance-level inter-frame association), and phenotypic measurement and analysis, together forming a scalable pipeline suitable for high-throughput applications. To ensure the fairness and reliability of the experiments, all experiments were independently repeated k=5 times under the same conditions, and the average results were reported to minimize randomness.

### 3.1 Real-time grain segmenter: YOLOv11-LA

#### 3.1.1 Image acquisition and detection data set construction

The data for this study were provided by the Rice Research Institute of the Jilin Academy of Agricultural Sciences. To generate sufficient phenotypic variation while keeping the genetic background constant, the same rice variety, “Tongjing 612” was cultivated under nine fertilization regimes that systematically combined straw incorporation, enzyme application, organic fertilizer, and chemical fertilizer (**Table 1**). Plot 1 received neither straw, enzyme, organic fertilizer, nor chemical fertilizer and therefore served as the unfertilized control. Plots 2 and 3 were designed to test the effect of straw incorporation with and without enzyme application. Plots 4–9 combined straw with different types of fertilizers: Plot 4 received all four inputs (straw, enzyme, organic and chemical fertilizers), Plots 5 and 6 received straw and enzyme in combination with either chemical or organic fertilizer, respectively, while Plots 7–9 received straw with different combinations of organic and/or chemical fertilizers but without enzyme. This design allowed us to disentangle the effects of straw, enzyme and fertilizer type on rice growth and phenotypic variation.

**Table 1:** Fertilization situation of each experimental plot.

Plot	Straw	Enzymes	Organic fertilizer	Chemical fertilizer
Plot 1	×	×	×	×
Plot 2	✓	✓	×	×
Plot 3	✓	×	×	×
Plot 4	✓	✓	✓	✓
Plot 5	✓	✓	×	✓
Plot 6	✓	✓	✓	×
Plot 7	✓	×	✓	×
Plot 8	✓	×	×	✓
Plot 9	✓	×	✓	✓

✓, use; ×, no use.

**Figure 2:**
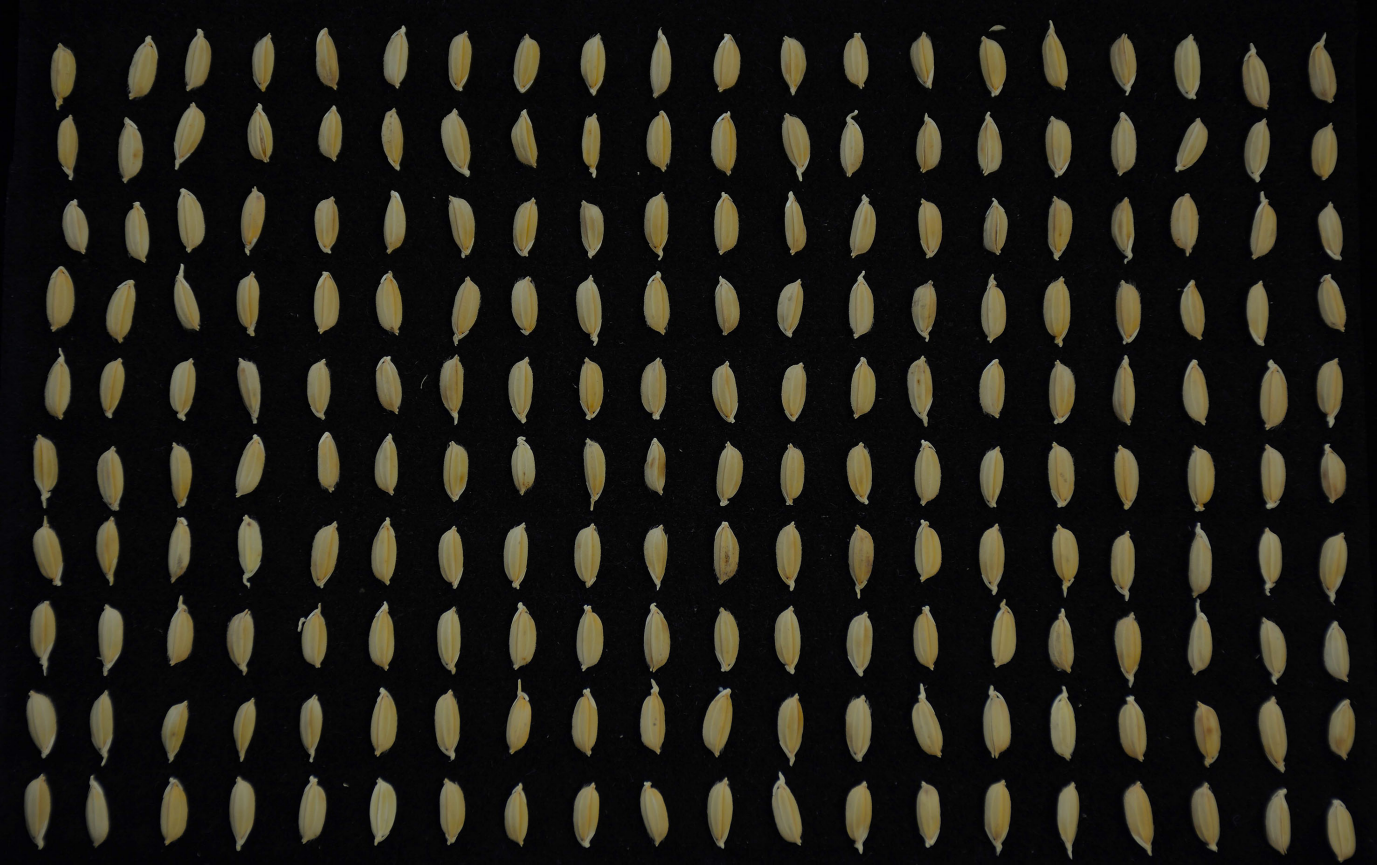
Rice seed photography rendering (6000×4000, 300dpi).

**Figure 3:**
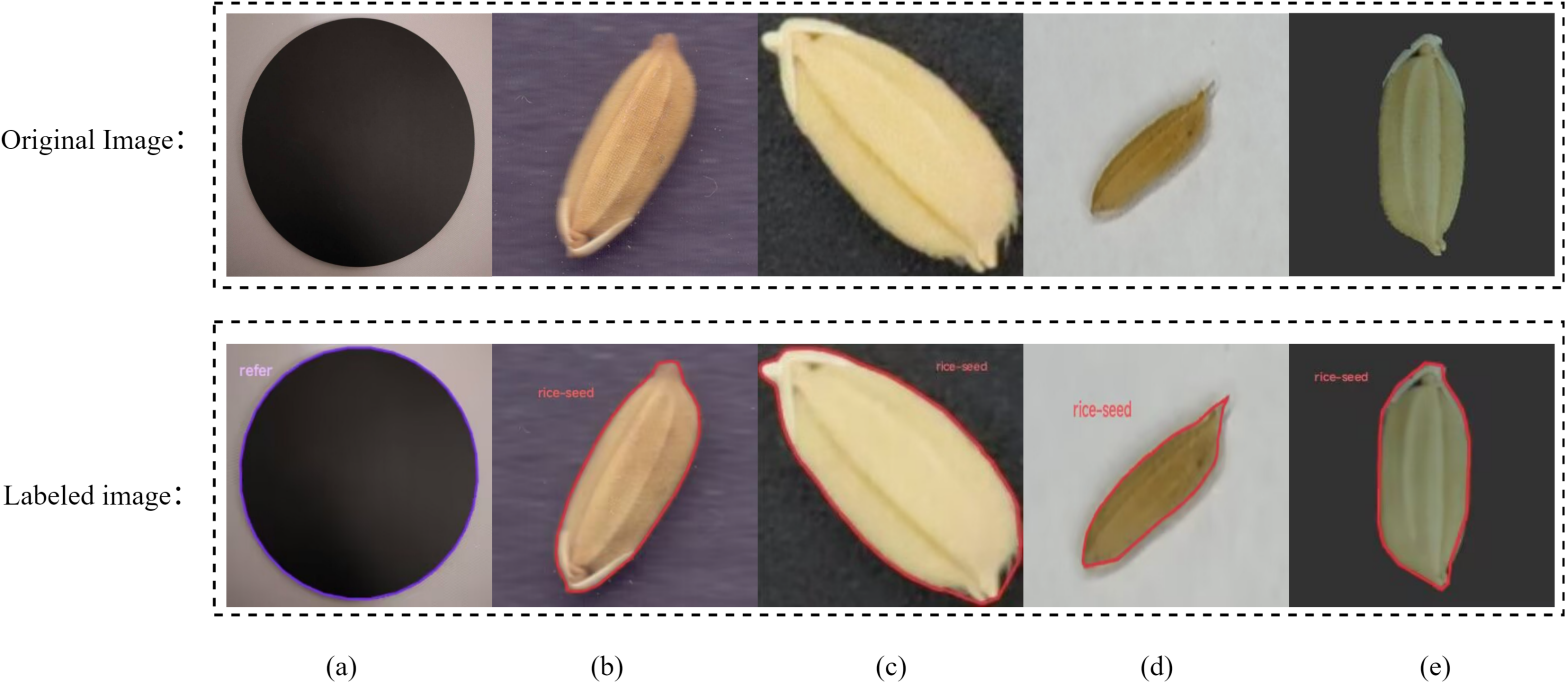
Example figure of multi-source datasets: **(a)** Absorbance reference plate; **(b)** RiceNet; **(c)** Japanese Rice; **(d)** RiceSeedSize; **(e)** Dataset used in this study.

Imaging was performed using a Nikon D7100 camera under uniform black background and standardized lighting conditions, with a resolution of 6000×4000 (300 dpi). Each image captured 200 grains; an example is shown in **Figure 2**. Additionally, we integrated public datasets including RiceNet (Mohi Ud Din et al., 2024), Japanese Rice (Rathnayake et al., 2023), and RiceSeedSize (Rajalakshmi et al., 2024) to enhance training data diversity and cross-scenario adaptability. To enable traceable pixel-to-physical-scale conversion, black light-absorbing discs were placed within images as dynamic scale calibration references. In this study, a total of 25,000 rice seed samples were collected and annotated. These were divided into training, validation, and test sets in an 8:1:1 ratio, comprising 20,000, 2,500, and 2,500 seeds, respectively. The training set was used for model parameter learning and feature extraction; the validation set was employed during training to tune hyperparameters and monitor convergence, preventing overfitting; the test set remained strictly independent from the model development process and was used only in the final stage for unified evaluation of the model’s generalization ability and overall performance, ensuring the objectivity and comparability of experimental results. All images were annotated with rice seed bounding boxes in YOLO format, forming the rice seed detection dataset. YOLO annotations for each dataset are illustrated in **Figure 3**.

#### 3.1.2 Network structure optimization of YOLOv11-LA

This study designs a lightweight segmentation model, YOLOv11-LA (Lightweight-Attention), based on the YOLOv11n architecture. Its primary optimizations include:

(1) Lightweight Convolution Structure Design: In the model backbone, the original two-layer standard convolution structure (Conv + Conv) is replaced with a Conv + DWConv combination. Depthwise Separable Convolution (DWConv) is also adopted in subsequent multi-layer feature extraction modules to reduce computational burden (Shafik et al., 2025). The computational complexity is optimized from standard convolution (Equation 1) to the depthwise separable structure (Equation 2):

(1) $$C_{\text{standard}}=K^{2}\cdot C_{\text{in}}\cdot C_{\text{out}}\cdot H\cdot W$$

(2) $$C_{\text{depthwise}}=K^{2}\cdot C_{\text{in}}\cdot H\cdot W+C_{\text{in}}\cdot C_{\text{out}}\cdot H\cdot W$$

where *K* is the convolution kernel size, *C*_in_ and *C*_out_ are the input/output channel numbers, and *H* × *W* is the output feature map size.

(2) Streamline the C3k2 module structure to reduce redundant computations by replacing deeper C3 variants with C3k2 ( k=2). Apply a compression coefficient γ∈(0,1] to the channel count at corresponding stages (while preserving the backbone resolution and receptive field) to reduce parameters and FLOPs. This modification inherits C3’s cross-stage feature fusion advantage (CSP mechanism) while significantly reducing redundant computations caused by stack depth and intermediate channels (Jocher et al., 2022).

(3) Introducing CBAM Attention Module before Detection Head (Woo et al., 2018) To highlight discriminative features of adhesions and minute grains at low cost, we insert CBAM once before the classification/regression convolutions in the detection head (channel attention with reduction ratio *r* = 16, spatial attention using 7×7 convolutions, as shown in the Equations 3, 4). This placement strategy focuses attention on high-level features after multi-scale fusion without overly interfering with the backbone’s general representations. This modification consistently improves performance while keeping GFLOPs largely unchanged.

(3) $$M_{c}(F)=\sigma (\text{MLP}(\text{AvgPool}(F))+\text{MLP}(\text{MaxPool}(F)))$$

(4) $$M_{s}(F)=\sigma (f^{7\times 7}([\text{AvgPool};\text{MaxPool}]))$$

Where AvgPool denotes Global Average Pooling, averaging the spatial dimensions (H,W) of feature map $F\in {\mathbb{R}}^{C\times H\times W}$ and outputting ${\mathbb{R}}^{C}$; MaxPool denotes Global Max Pooling, which takes the maximum value in the spatial dimensions (H,W) and outputs ${\mathbb{R}}^{C}$; MLP represents a two-layer fully connected network (typically with ReLU activation in the middle layer), used to generate channel attention weights; $f^{7\times 7}$ denotes a convolution operation with a 7×7 kernel, taking 2 input channels (concatenated from [AvgPool;MaxPool]) and producing 1 output channel; σ(·) denotes the Sigmoid activation function, which compresses values into the (0,1) range; [· ; ·] represents concatenation along the channel dimension.

(4) Channel width reduction: The maximum output channel count of the backbone is reduced from 1024 to 512. The output channels of P5 in the Head module are also simplified accordingly, effectively compressing the parameter scale and reducing memory consumption.

**Figure 4:**
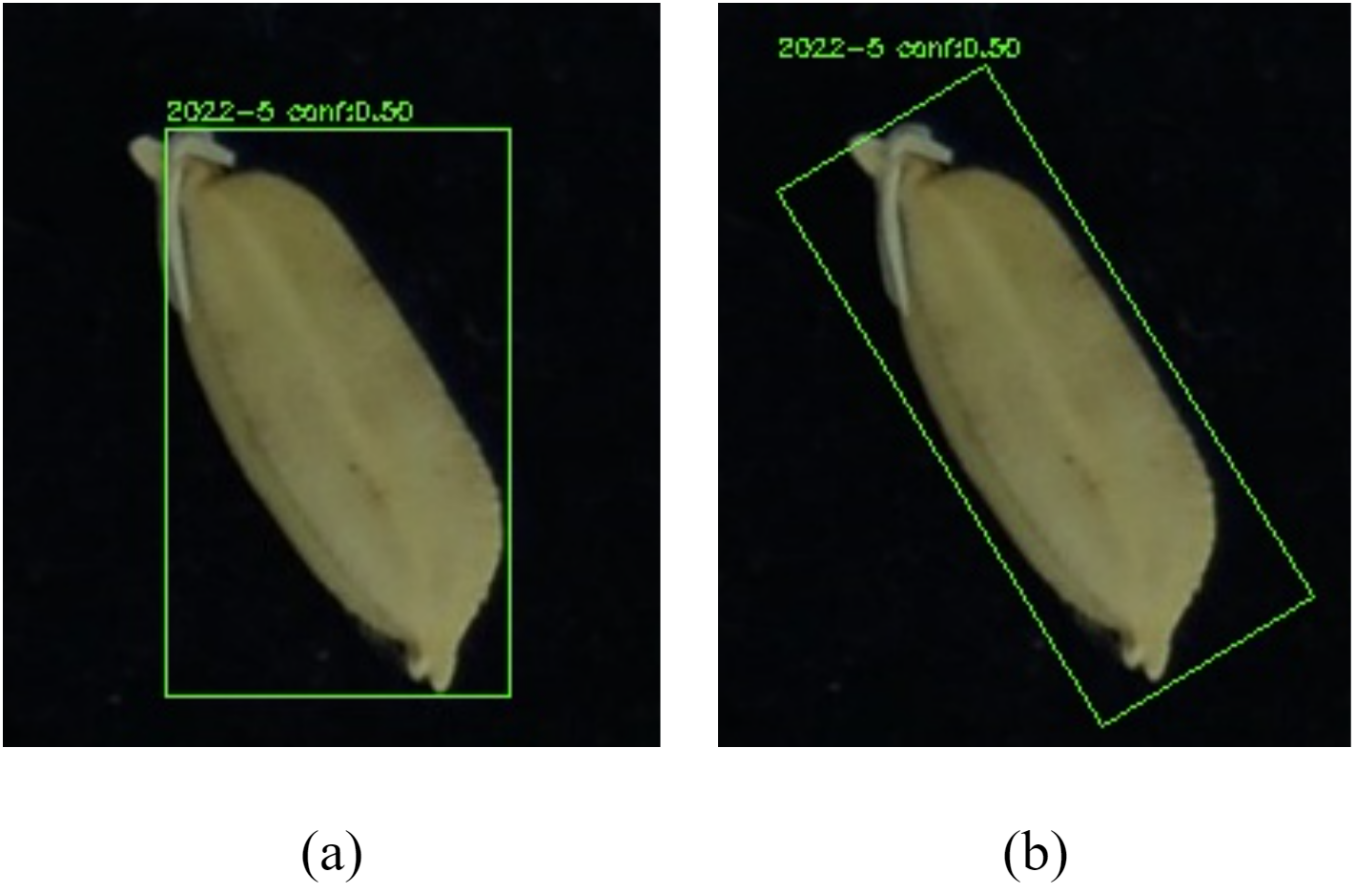
**(a)** Original YOLO detection box; **(b)** Minimum bounding rectangle detection box after applying Otsu.

**Figure 5:**
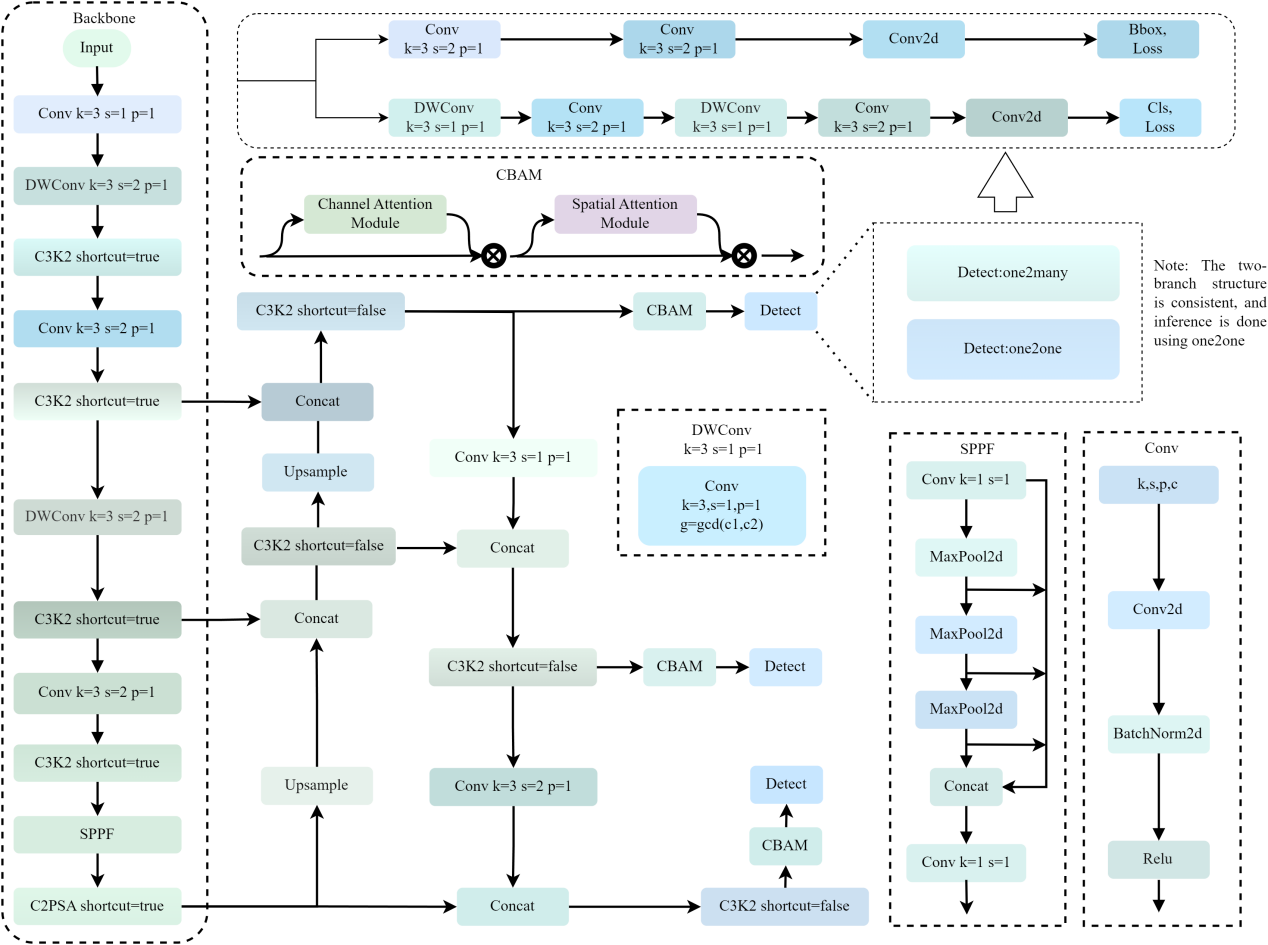
YOLOv11-LA network structure.

Furthermore, to address the limitation of conventional YOLO boxes failing to accurately fit tilted seed grain boundaries, the Otsu adaptive thresholding method is introduced. This performs fine segmentation on each detected seed grain region and extracts the minimum bounding rectangle, thereby enhancing the accuracy of subsequent phenotypic measurements. **Figure 4** demonstrates the effect of using minimum bounding rectangles, and the model structure diagram is shown in **Figure 5**.

### 3.2 Rice seed classifier: RiceLCNN

#### 3.2.1 Classification dataset construction

**Figure 6:**
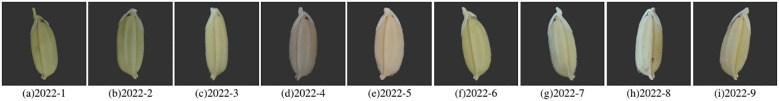
Rice seed samples harvested from plots with different fertilization treatments (Plots 1–9). The seed samples, collected in 2022, vary in grain shape, surface texture, and color based on the application combinations of straw return (S), enzymes (E), organic fertilizer (O), and chemical fertilizer (C). These differences provide distinct phenotypic features for image-based intelligent segmentation and classification. **(a)** Rice seeds from Plot 1, **(b)** Rice seeds from Plot 2, **(c)** Rice seeds from Plot 3, **(d)** Rice seeds from Plot 4, **(e)** Rice seeds from Plot 5, **(f)** Rice seeds from Plot 6, **(g)** Rice seeds from Plot 7, **(h)** Rice seeds from Plot 8, **(i)** Rice seeds from Plot 9.

Based on the detection outputs from YOLOv11-LA, each rice seed image was automatically cropped and uniformly resized to 224 × 224 pixels. After removing occluded, blurred, and incorrectly detected images, a high-quality rice seed classification dataset comprising 16,731 images was ultimately constructed. The data was partitioned into training, validation, and test sets at an 8:1:1 ratio, with specific counts shown in the **Table 2**. The naming convention reflects the collection year and experimental plot number. Grain samples of each variety are displayed in **Figure 6**.

**Table 2:** Distribution of training, validation, and test data for nine rice varieties.

Rice variety	No. of training samples	No. of validation samples	No. of test samples
2022-1	1,487	186	186
2022-2	1,487	186	186
2022-3	1,487	186	186
2022-4	1,487	186	186
2022-5	1,487	186	186
2022-6	1,487	186	186
2022-7	1,487	186	186
2022-8	1,487	186	186
2022-9	1,487	186	186
Total	13,383	1,674	1,674

#### 3.2.2 RiceLCNN network structure design

**Figure 7:**
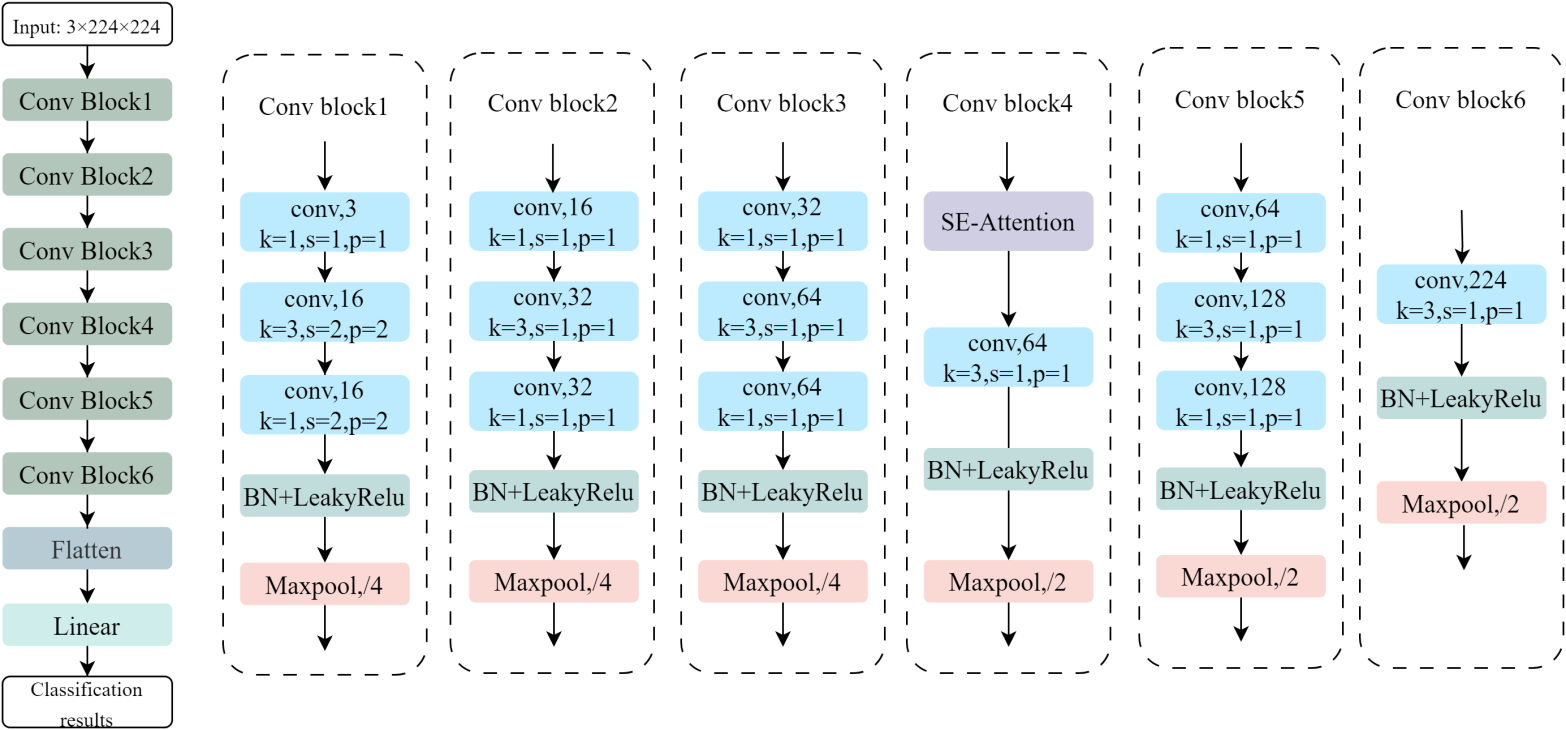
RiceLCNN network structure.

To balance inference efficiency on mobile devices with rice seed classification accuracy, this paper proposes the lightweight convolutional neural network RiceLCNN. The model backbone consists of six cascaded convolutional modules followed by a fully connected classification head (**Figure 7**), adhering to the lightweight paradigm of “1 × 1 bottleneck restructuring + 3 × 3 local modeling.” Batch Normalization and LeakyReLU are applied after all convolutions to stabilize gradients and accelerate convergence (Bao et al., 2025; Wang et al., 2019). The first three modules employ a structure composed of sequential 1×1, 3×3, and 1×1 convolutions. The initial stage performs early downsampling using a stride *s* = 2 at the 3 × 3 convolution, followed by further spatial dimension reduction via 4 × 4 max pooling. The second and third stages maintain convolutions with stride *s* = 1 and similarly employ 4 × 4 max pooling to control resolution. After these three stages, the backbone introduces channel attention to enhance discriminative feature representation: Specifically, a Squeeze-and-Excitation (SE) mechanism is applied to the 64-channel features for channel recalibration. First, global average pooling is performed on the feature *F* to obtain the channel description *z* = GAP(*F*). Then, two fully connected layers with nonlinear transformations generate the weight vector, as shown in the following Equation 5.

(5) $$s=\sigma \;(W_{2}\;\delta (W_{1}z))$$

where *δ*(·) and *σ*(·) denote ReLU and Sigmoid, respectively. *s* is used for per-channel scaling of the original features.

Following SE, a 3 × 3 convolution layer and 2 × 2 max pooling continue feature refinement and compression. The fifth stage reuses the 1×1, 3×3, 1×1 convolution sequence followed by 2 × 2 pooling downsampling. The sixth stage employs a single 3×3 convolution for final semantic aggregation, followed by 2×2 pooling to obtain a compact spatial representation. Channel sizes follow a monotonically increasing pyramidal configuration along the network backbone: {16, 32, 64, 64, 128, 224}. Here, 1 × 1 convolutions perform cross-channel feature reorganization and dimension reduction at low cost, while 3×3 convolutions focus on critical local contexts. The SE module adaptively reweights channel importance during the mid-level semantic stage, enhancing feature selection capability and inter-class separability within limited parameter and computational budgets (Ding et al., 2021; Li et al., 2020; Liu et al., 2023). Finally, the network obtains a 224-dimensional global representation through a flattening operation and outputs classification results via a single fully-connected layer (224 × *C*, where *C* denotes the number of classes). This design effectively improves recognition performance while maintaining manageable inference overhead, making it suitable for resource-constrained mobile deployment scenarios.

### 3.3 Integrated tracking counting and morphometry module

#### 3.3.1 Real-time tracking and counting

**Figure 8:**
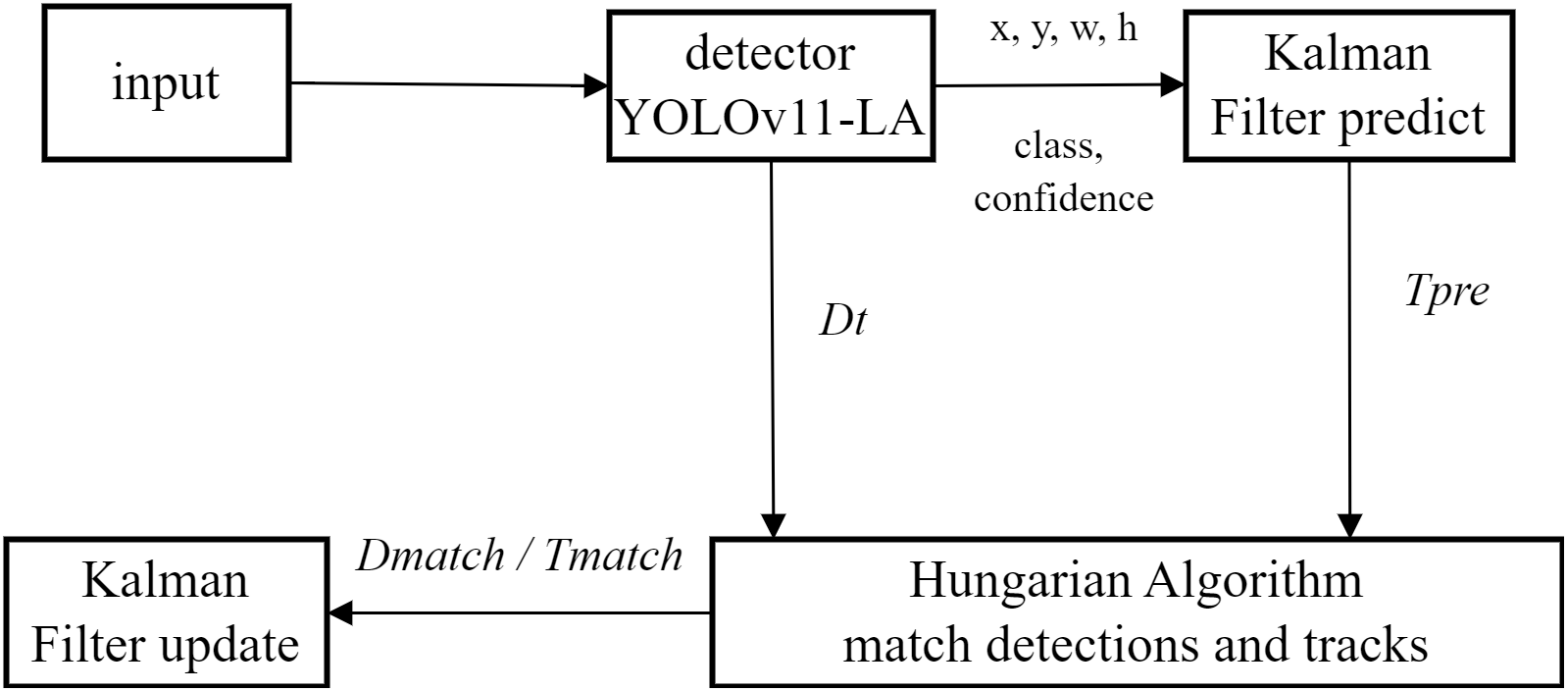
Flowchart of YOLOv11-LA Integrating DeepSORT.

In the scenario of simultaneous multi-grain rice variety identification, we employ YOLOv11-LA as the front-end detector. For each frame image *I_t_*, it outputs a detection set $D_{t}={\left\{(x_{i},y_{i},w_{i},h_{i},c_{i},s_{i})\right\}}_{i=1}^{N_{t}}$, where (x,y,w,h) denotes the center and scale of the bounding box, $c_{i}$ represents the initial category (rice-seed), and $s_{i}$ indicates the confidence score. The detection results are fed into DeepSORT for cross-frame association: for each in-orbit target $T_{k}$, establish a Kalman filter state $x_{k}={\left[u,v,\gamma ,h,\dot{u},\dot{v},\dot{\gamma },\dot{h}\right]}^{⊤}$ (where u,v are the bounding box center, γ=w/h), yielding the prior trajectory set ${\text{T}}_{t}^{\text{pre}}$. Construct the cost matrix C under the threshold of Mahalanobis distance and IoU. Solve the global optimal matching of $\left(D_{t},{\text{T}}_{t}^{\text{pre}}\right)$ using cascade matching (starting from the most recent update time of the trajectory and proceeding from near to far) and the Hungarian algorithm, obtaining the set of matching pairs $M_{t}$. Perform Kalman updates on $(d_{i},T_{k})\in M_{t}$ to obtain the corrected trajectory ${\text{T}}_{t}^{\text{match}}$. Unmatched detections initialize new trajectories, and trajectories unmatched for more than ${\text{Δ}}_{\text{max}}$ consecutive frames are terminated. This dual “cascade + IoU” constraint effectively reduces missed detections and ID switching (Yan et al., 2025), as 272 illustrated in **Figure 8**.

For each confirmed track, the cropped region $I_{t}^{\left(k\right)}=\text{crop}(I_{t},T_{k})$ is input to the lightweight classifier RiceLCNN to obtain category prediction ${\hat{y}}_{t}^{\left(k\right)}=\text{arg }\max_{y}p(y\;|\;I_{t}^{\left(k\right)})$ and confidence score ${\hat{s}}_{t}^{\left(k\right)}$. Combined with dynamic scale calibration, this outputs phenotypic measurements including length, width, aspect ratio, and roundness. Counting employs a unique ID deduplication strategy: Let $U_{t}$ denote the set of confirmed trajectories first entering the counting region by time t that remain uncounted. The cumulative count is then $N_{t}=N_{t-1}+|U_{t}|$. In dense images with coalescing but minimal occlusion, this modular “detection-tracking-classification-measurement” pipeline maintains consistent cross-frame IDs and stable counting. Key parameters of DeepSORT are shown in **Table 3**, with ID switches (IDSW) in the test sequence reduced to 2.1(average).

**Table 3:** Hyperparameter configuration of the improved DeepSORT tracking module.

Parameter	Value	Description
max_dist	0.2	Cosine similarity threshold for detection-tracklet matching
min_confidence	0.3	Filters low-confidence detections to reduce noise
nms_max_overlap	0.5	Removes duplicate detections in dense areas
max_iou_distance	0.7	Spatial matching tolerance in Hungarian algorithm
max_age	70	Frames to retain lost targets before deletion
n_init	3	Required consecutive matches for new track initiation
nn_budget	100	Historical feature queue length stored per tracker
num_target	751	Number of trackable objects in the dataset

#### 3.3.2 Real-time measurement

To achieve dynamic absolute scale conversion based on the image, this paper directly employs YOLOv11- LA for detection and segmentation of the calibrated disk: The detector first outputs the disk’s Region of Interest (ROI) (category scale disk), and its segmentation head returns the disk mask $M_{d}$ within this ROI. The pixel domain area is obtained by counting pixels in the mask, as shown in the following Equation 6.

(6) $$A_{\text{px}}^{\left(d\right)}\;=\;\sum_{\left(i,j\right)}1[\;(i,j)\in {\text{M}}_{d}],$$

where $A_{\text{px}}^{\left(d\right)}$ denotes the pixel-domain area of the disk (units: ${\text{px}}^{2}$), 1[·] is the idempotent function, and (i,j) represents pixel coordinates.

Based on the circle area-diameter relationship, the equivalent pixel diameter of the disk is obtained as shown in the following Equation 7.

(7) $$D_{\text{pix}}\;=\;2\sqrt{\frac{A_{\text{px}}^{\left(d\right)}}{\pi }},$$

where *D*_pix_ denotes the diameter in the pixel domain (unit: px). This formula leverages mask geometry without requiring additional edge/ellipse fitting, making it suitable for real-time high-throughput applications.

Comparing the pixel diameter to the physical diameter yields a uniform millimeter/pixel scale across the entire image, as shown in the following Equation 8.

(8) $$\alpha \;=\;\frac{D_{\text{real}}}{D_{\text{pix}}}\;[\text{mm}/\text{px}],$$

where *D*_real_ is the true diameter of the calibrated disk (unit: mm), and *α* is the scale factor mapping lengths from the pixel domain to the physical domain. This paper assumes near-orthogonal imaging with square (isotropic) pixels; if minor perspective/distortion exists, camera calibration is applied for prior correction.

After obtaining α, for each detected seed instance (category seed), contours are extracted within its detection bounding box using the Otsu method. These are then processed through Gaussian smoothing, Sobel gradient detection, and sub-pixel edge fitting to derive the pixel-domain metrics $\left(L_{\text{px}},W_{\text{px}},A_{\text{px}}^{\left(s\right)},P_{\text{px}}\right)$ within its detection bounding box. The corresponding physical domain quantities are converted according to the following Equation 9:

(9) $$L_{\text{mm}}=\alpha \;L_{\text{px}},\;W_{\text{mm}}=\alpha \;W_{\text{px}},\;A_{{\text{mm}}^{2}}=\alpha^{2}A_{\text{px}}^{\left(s\right)},\;P_{\text{mm}}=\alpha \;P_{\text{px}},$$

where $L_{\text{mm}},W_{\text{mm}}$ denote seed length and width (mm), $A_{{\text{mm}}^{2}}$ denotes area ( ${\text{mm}}^{2}$), and $P_{\text{mm}}$ denotes perimeter (mm); The superscript (*s*) denotes seed-related quantities.

Roundness is a scale-invariant property and can be computed equivalently in both the pixel domain and the physical domain. The calculation process is shown in Equation 10.

(10) $$C\;=\;\frac{4\pi A}{P^{2}}\;=\;\frac{4\pi A_{px}^{\left(s\right)}}{P_{\text{p}x}^{2}}\;=\;\frac{4\pi A_{\text{m}{\text{m}}^{2}}}{P_{\text{mm}}^{2}},$$

where *A,P* must be taken from the same measurement domain; *C* reflects the degree of circularity of the contour (*C* ∈ (0,1]).

To evaluate measurement uncertainty, this paper employs a first-order error propagation approximation, as shown in Equation 11.

(11) $$\text{Δ}L_{\text{mm}}\;\approx \;\sqrt{{\left(\hat{\alpha }\text{ Δ}L_{\text{px}}\right)}^{2}\;+\;{\left(L_{\text{px}}\;\text{Δ}\hat{\alpha }\right)}^{2}},$$

where $\hat{\alpha }$ is the estimated scale, $\text{Δ}L_{\text{px}}$ is the pixel-domain length error caused by sub-pixel edge localization, and $\text{Δ}\hat{\alpha }$ is the standard deviation of the scale estimate. This equation quantifies the combined uncertainty contribution from segmentation boundary and scale estimation noise to the physical-domain length.

## 4 Experiments

### 4.1 Experimental environment and parameters

This experiment leverages the PyTorch framework and utilizes a Quadro RTX 8000 GPU (48 GB VRAM, 672 GB/s bandwidth, 4608 CUDA cores) under CUDA 12.6 for accelerated training. The segmentation model uses a uniform input size of 1080 × 720 with a batch size of 8. The optimizer operates in auto mode with an initial learning rate of 0.01. The classification model employs an input size of 3 × 224 × 224 with a batch size of 64, trained for 50 epochs. The optimizers include SGD and Adam, with an initial learning rate of 0.001.

### 4.2 Experimental evaluation metrics

To comprehensively evaluate the performance of the proposed model in rice seed detection and classification tasks, this paper constructs a multidimensional evaluation metric system focusing on accuracy, robustness, and computational complexity. The detection task primarily assesses the model’s ability to accurately identify the location of rice seed targets, while the classification task emphasizes the model’s capability to distinguish between different rice seed varieties or grades. All evaluation metrics are calculated based on an independent test set, with specific definitions and calculation formulas as follows.

(1) Model Complexity: To measure model lightweight nature and computational overhead, the following metrics are calculated: Parameters (Params): Total number of trainable parameters in the model; GFLOPs (Giga Floating Point Operations per Second): The floating-point computation required for forward inference on a single image, as shown in Equation 12.

(12) $$\text{GFLOPs}=\frac{\sum_{l=1}^{L}2\cdot C_{l}^{in}\cdot K_{l}^{2}\cdot C_{l}^{out}\cdot H_{l}\cdot W_{l}}{10^{9}}$$

where *C_l_*^in^ and *C_l_*^out^ denote the input and output channel counts of the *l*th convolutional layer, respectively; *K_l_*is the convolution kernel size; *H_l_*× *W_l_*represents the spatial resolution of the feature map at that layer; and *L* is the total number of convolutional layers.

(2)Object detection metrics: The quality of bounding box predictions is evaluated using three metrics—Precision (*P*), Recall (*R*), and Mean Average Precision (mAP) (Wen et al., 2025). The calculation processes are shown in Equations 13–15, respectively.

(13) $$P=\frac{TP}{TP+FP}\;$$

(14) $$R=\frac{TP}{TP+FN}\;$$

In Equation 13, *TP* denotes the number of samples predicted as positive and actually positive (true positives), while *FP* denotes the number of samples predicted as positive but actually negative (false positives); In Equation 14, *FN* denotes missed samples (false negatives). For detection tasks, the definitions of *TP*, *FP*, and *FN* depend on whether the Intersection over Union (IoU) between predicted and ground-truth bounding boxes exceeds a set threshold (e.g., 0.5).

(15) $$\text{mAP}=\frac{1}{N}\sum_{i=1}^{N}\int_{0}^{1}P_{i}(R_{i})\;\text{d}R_{i}\;$$

where *N* is the total number of target classes, and *P_i_*(*R_i_*) denotes the precision-recall (PR) curve for the *i*th class. This paper reports two metrics: mAP@0.5 (IoU threshold of 0.5) and mAP@0.5:0.95 (averaging samples from 0.5 to 0.95), reflecting detection performance under lenient and stringent conditions, respectively.

(3) Image Classification Metrics: To evaluate the model’s discriminative capability for rice variety classification, we introduce Accuracy and F1 Score [where Precision (*P*) and Recall (*R*) are defined in (13) and (14)]. Accuracy is defined as shown in Equation 16.

(16) $$\text{Accuracy}=\frac{TP+TN}{TP+TN+FP+FN}\;$$

where *TN* denotes the number of samples correctly classified as negative. This metric measures the proportion of correctly classified samples across all categories. To further evaluate the model’s performance in positive class recognition, the F1 score is introduced as shown in Equation 17.

(17) $$F_{1}=\frac{2\cdot P\cdot R}{P+R}\;$$

F1 combines precision and recall, serving as their harmonic mean and being well-suited for evaluating models in scenarios with class imbalance. For multi-class classification tasks, the macro-average strategy is employed: *P*, *R*, and *F*_1_ are calculated separately for each class, and their arithmetic mean is taken to fairly reflect the importance of each class. Distinction: While Precision and Recall share identical calculation methods, their meanings and emphasis differ across tasks: In object detection, these metrics evaluate how well detection boxes overlap with targets, prioritizing spatial accuracy and false negative rates. In classification tasks, they measure a model’s ability to distinguish between categories, particularly crucial for identifying intermediate, adjacent classes.

### 4.3 YOLOv11-LA lightweight model improvement and ablation experiments

To reduce computational and memory overhead while maintaining detection accuracy, this paper proposes the lightweight detection network YOLOv11-LA based on the YOLOv11n baseline. The design focuses on four orthogonal structural optimizations: (i) replacing part of the standard convolutions with Depthwise Separable Convolution (DWConv+PWConv) to reduce parameters and FLOPs; (ii) streamlining the C3k2 feature extraction unit and simultaneously narrowing channel width to suppress redundant computation (Slim); (iii) introducing CBAM attention in the detection head to enhance the representation of key regions; and (iv) imposing a maximum-channel cap (512 channels) on the widest layers to further control model size.

To quantify how each strategy contributes to the performance–efficiency trade-off, we constructed seven ablation configurations (**Table 4**). A denotes the original YOLOv11n baseline. B (YOLOv11n-DW) isolates the effect of DWConv by only replacing the corresponding standard convolutions in A. C (YOLOv11n-Slim) keeps the convolution type unchanged but simplifies the C3k2 blocks and compresses channels, so that the impact of structural slimming on accuracy and complexity can be observed independently. D (YOLOv11n-Attn) inserts CBAM modules into the detection head on top of A, leaving the backbone unchanged, which isolates the contribution of attention. E (YOLOv11n-SlimAttn) combines the Slim backbone in C with the attention mechanism in D to investigate the interaction between compression and attention under a low-computation regime. F (YOLOv11n-Cap) applies only the maximum-channel cap to the baseline A, without DWConv, Slim, or attention, to isolate the influence of the channel cap itself on model capacity and efficiency. Finally, G (YOLOv11-LA) integrates all the above optimizations—DWConv, Slim C3k2 blocks with channel compression, CBAM attention, and the channel cap—to obtain the final lightweight model.

**Table 4:** Ablation study results of different configurations of YOLOv11-LA on rice seed detection (values reported as mean ± std).

Algorithm	P (%)	R (%)	mAP@0.5	mAP@0.5:0.95	GFLOPs	Parameters
A(YOLOv11n)	98.83 ± 0.23	99.55 ± 0.19	99.50 ± 0.12	91.16 ± 0.30	6.4	2,590,230
B(YOLOv11n-DW)	98.51 ± 0.24	99.37 ± 0.22	99.50 ± 0.14	90.69 ± 0.31	5.8	2,439,606
C(YOLOv11n-Slim)	97.60 ± 0.28	99.07 ± 0.26	98.43 ± 0.20	82.09 ± 0.48	3.3	942,814
D(YOLOv11n-Attn)	99.79 ± 0.12	99.72 ± 0.15	99.49 ± 0.12	92.89 ± 0.27	6.5	2,569,434
E(YOLOv11n-SlimAttn)	98.38 ± 0.23	99.46 ± 0.21	99.45 ± 0.13	92.41 ± 0.29	3.4	992,994
F(YOLOv11n-Cap)	97.10 ± 0.11	98.50 ± 0.12	99.40 ± 0.10	92.40 ± 0.13	3.3	942,789
G(YOLOv11-LA)	99.15 ± 0.18	99.67 ± 0.16	99.50 ± 0.12	93.06 ± 0.25	3.1	953,682

As shown in **Table 4**, B significantly reduces computational and parameter overhead (GFLOPs drop from 6.4 to 5.8, i.e., a 9.4% reduction; parameters decrease from 2.59M to 2.44M) with almost no loss in detection accuracy (mAP@0.5 remains at 99.50%). C further compresses the model to 0.94M parameters and 3.3 GFLOPs (48.4% fewer FLOPs than A), but causes a noticeable drop in mAP@0.5:0.95 (from 91.16% to 82.09%), indicating that overly aggressive structural slimming alone harms localization and scale regression of dense small objects. D shows that CBAM attention mainly improves accuracy: compared with A, YOLOv11n-Attn slightly increases complexity (GFLOPs from 6.4 to 6.5), but boosts mAP@0.5:0.95 by 1.73 percentage points (from 91.16% to 92.89%), confirming that attention is beneficial even without compression.

The additional configuration F highlights the isolated effect of the channel cap. Compared with the baseline A, YOLOv11n-Cap achieves a substantial reduction in complexity (GFLOPs from 6.4 to 3.3, i.e., a 48.4% reduction; parameters from 2.59M to 0.94M, a 63.6% reduction), while maintaining high detection performance: mAP@0.5 only decreases slightly (from 99.50% to 99.40%), and mAP@0.5:0.95 even improves from 91.16% to 92.40%. Under a similar computational cost to C, F therefore provides much better overall detection quality (mAP@0.5:0.95 improves from 82.09% to 92.40%), indicating that redistributing capacity via a channel cap is more favorable than pure structural slimming.

The combined configurations E and G further reveal the interaction among these modules. Adding CBAM to the slimmed model (E) recovers most of the accuracy loss of C: mAP@0.5:0.95 is increased from 82.09% to 92.41%, while GFLOPs remain at only 3.4 and parameters below 1.0M. On this basis, integrating DWConv and the channel cap in G further improves the trade-off: YOLOv11-LA achieves mAP@0.5 of 99.50% and mAP@0.5:0.95 of 93.06% with only 3.1 GFLOPs and 0.95M parameters, corresponding to a 51.6% reduction in computation and about a 63.2% reduction in parameters compared with the baseline A, while also increasing mAP@0.5:0.95 by 1.90 percentage points. These results demonstrate that under dense, small-object and occlusion-prone scenarios, each lightweighting strategy contributes in a complementary way, and their combination in YOLOv11-LA maintains or even improves detection accuracy while significantly reducing model size and computational cost, making it well-suited for edge deployment.

### 4.4 Comparison experiment design

#### 4.4.1 Detection model comparison experiment

To comprehensively evaluate the performance of the proposed lightweight improved network YOLOv11-LA, we trained and validated five object detection models on the rice seed detection dataset: YOLOv5n, YOLOv8n, YOLOv10n, YOLOv11n, and the enhanced YOLOv11-LA. All models were trained with identical parameter settings and underwent three independent experimental runs to ensure result stability and reproducibility. Experimental results are shown in **Table 5**.

**Table 5:** Comparison of results from each model in the contrast experiment (values reported as mean ± std).

Metric	YOLOv5n	YOLOv8n	YOLOv10n	YOLOv11n	YOLOv11-LA
Precision	98.43 ± 0.22	98.00 ± 0.25	97.31 ± 0.27	98.83 ± 0.23	99.15 ± 0.18
Recall	98.59 ± 0.24	98.82 ± 0.22	95.55 ± 0.35	99.55 ± 0.19	99.67 ± 0.16
mAP@0.5	99.42 ± 0.14	99.47 ± 0.13	99.05 ± 0.16	99.55 ± 0.12	99.50 ± 0.12
mAP@0.5:0.95	91.37 ± 0.28	93.06 ± 0.26	91.81 ± 0.30	91.16 ± 0.30	93.06 ± 0.25
GFLOPs	7.2	8.2	8.4	6.4	3.1
Parameters	2,508,854	3,011,238	2,707,820	2,590,230	953,682

As shown in **Table 5**, YOLOv11-LA demonstrates superior performance at both common IoU thresholds, outperforming all comparison models. At IoU=0.5, YOLOv11-LA achieves mAP improvements of 0.08%, 0.03%, 0.45%, and -0.05% compared to YOLOv5n, YOLOv8n, YOLOv10n, and YOLOv11n, respectively. Although numerically close to YOLOv11n, YOLOv11-LA demonstrates a more significant advantage under the stricter mAP@0.5:0.95 metric, achieving improvements of 1.69%, 1.25%, and 1.9% over YOLOv5n, YOLOv10n, and YOLOv11n, respectively. Indicating its enhanced robustness across multiple scales and boundary conditions.

In computational complexity, YOLOv11-LA achieves 3.1 GFLOPs, reducing computational load by approximately 56.9%, 62.2%, 63.1%, and 51.6% compared to YOLOv5n, YOLOv8n, YOLOv10n, and YOLOv11n, respectively. Simultaneously, YOLOv11-LA demonstrates significant advantages in model parameter count. With 953,682 parameters, it represents only 38% of YOLOv5n (2,508,854), 31.7% of YOLOv8n (3,011,238), YOLOv10n (2,707,820) by 35.2%, and YOLOv11n (2,590,230) by 36.8%. This compression ratio of approximately 2.5 to 3 times significantly reduces memory consumption, demonstrating robust, lightweight characteristics and strong potential for edge deployment.

**Figure 9:**
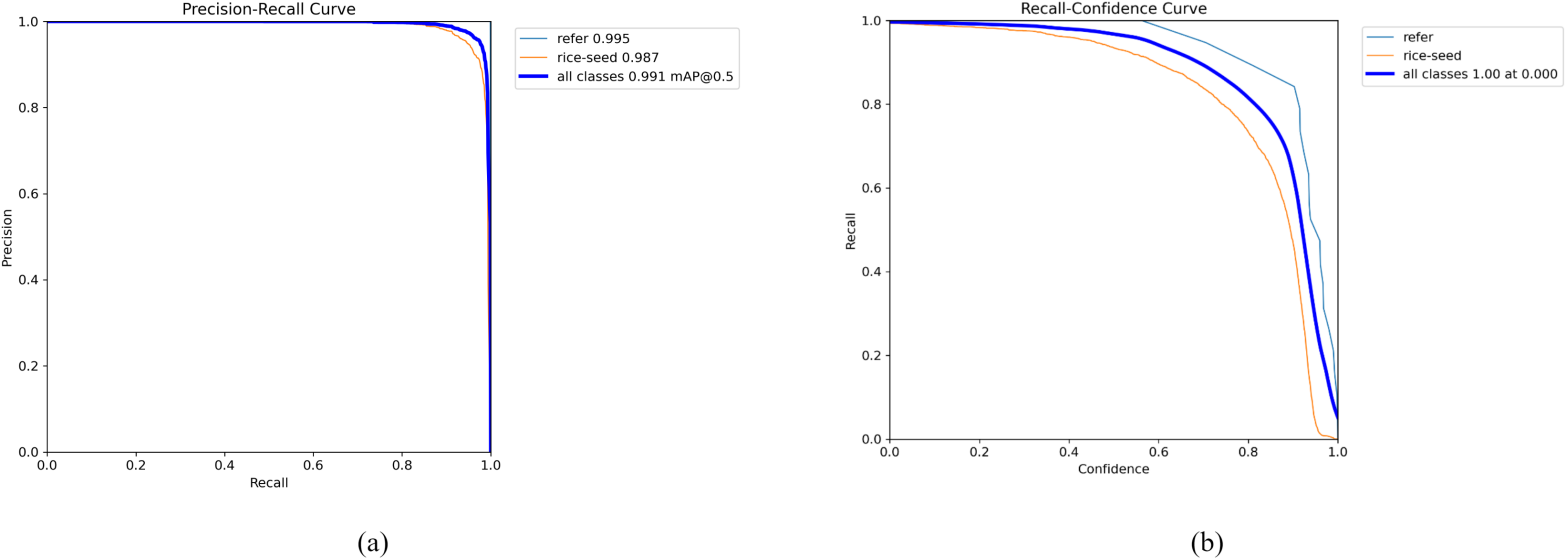
**(a)** Precision-Recall curve of YOLOv11-LA; **(b)** Recall-Confidence curve of YOLOv11-LA.

Although YOLOv8n exhibits good accuracy on certain categories, its parameter count and computational cost are significantly higher than YOLOv11-LA. Meanwhile, YOLOv11-LA achieves higher detection accuracy on most key categories while maintaining low complexity, demonstrating an excellent performance-complexity tradeoff. **Figure 9** displays the Precision-Recall curve and Recall-Confidence curve of the YOLOv11-LA model on the rice seed detection task. The model demonstrates outstanding performance across both categories (refer and rice-seed), achieving peak accuracies of 0.995 and 0.987, respectively. Its overall mAP@0.5 reaches 0.991, indicating exceptional discrimination capabilities in multi-class object recognition.

#### 4.4.2 Classification model comparison experiment

To comprehensively evaluate the classification performance of the RiceLCNN model, this study compared it against current mainstream deep learning classification models, including MobileNetV2 (Model A), MobileNetV3 (Model B, using small variant), Xception (Model C), ResNet50 (Model D), EfficientNetV2 (Model E), ShuffleNetV2 (Model G, using 0.5x variant) and Vision Transformer (Model H, using ViT-B/16). RiceLCNN was evaluated as the comparison model F. Specific comparison results include the number of parameters, training speed (seconds per epoch, abbreviated as “Speed”), classification accuracy (Accuracy, abbreviated as “Acc”), precision (P), recall (R), and F1 score for each model, as shown in **Table 6**.

**Table 6:** Comparison results of various classification models (values reported as mean ± std).

Algorithm	Params	Speed (s/Ep.)	Acc (%)	P (%)	R (%)	F1 (%)
A (MobileNetV2)	3,504,872	30.23 ± 0.41	85.78 ± 0.32	85.78 ± 0.33	86.53 ± 0.34	85.82 ± 0.31
B (MobileNetV3)	2,542,856	25.23 ± 0.38	87.77 ± 0.34	85.78 ± 0.33	86.53 ± 0.34	85.82 ± 0.31
C (Xception)	20,045,897	40.12 ± 0.47	88.77 ± 0.35	88.77 ± 0.34	88.95 ± 0.33	88.82 ± 0.32
D (ResNet50)	24,045,897	75.00 ± 0.50	87.28 ± 0.33	87.28 ± 0.32	87.57 ± 0.33	87.34 ± 0.32
E (EfficientNetV2)	19,045,897	70.21 ± 0.49	85.66 ± 0.30	85.66 ± 0.31	86.09 ± 0.32	85.75 ± 0.31
F (RiceLCNN)	527,614	15.19 ± 0.29	89.78 ± 0.36	89.78 ± 0.35	89.93 ± 0.34	89.81 ± 0.33
G (ShuffleNetV2)	352,042	14.03 ± 0.28	84.77 ± 0.30	85.88 ± 0.33	84.77 ± 0.30	84.93 ± 0.31
H (Vision Transformer)	85,806,346	240.03 ± 20	65.89 ± 0.32	65.70 ± 0.33	65.89 ± 0.30	65.76 ± 0.31

**Table 6** shows that RiceLCNN achieves the highest prediction accuracy (89.78%) among all comparison models, demonstrating outstanding classification performance. With only 527,913 parameters, RiceLCNN is considerably more lightweight than large-scale models such as ResNet50 (24,045,897) and Xception (20,045,897), giving it a pronounced advantage in resource efficiency. In terms of training efficiency, RiceLCNN also performs exceptionally well, completing a single training cycle in just 15.19 seconds—approximately 39.8% faster than MobileNetV3-small (25.23 seconds)—further highlighting its effectiveness. By contrast, the Vision Transformer (ViT-B/16, Model H) exhibited substantially poorer classification performance than all CNN-based counterparts, achieving only 65.89% accuracy—significantly lower than RiceLCNN (89.78%) and even lightweight CNN models such as MobileNetV3 (87.77%).Despite having the largest representation capacity with 85.8M parameters, ViT was markedly less efficient, requiring about 240.03 ± 20 seconds per epoch, which is over 15 times slower than RiceLCNN. The inferior performance of ViT can be explained by several factors. First, Vision Transformers generally demand large-scale datasets to fully exploit their self-attention mechanism, while the rice seed dataset used in this study is relatively limited in both size and diversity. This makes ViT more susceptible to underfitting or unstable feature learning, especially in fine-grained classification tasks such as distinguishing visually similar seed categories. Second, unlike CNNs, ViT lacks strong inductive biases (e.g., locality and translation equivariance) that are particularly advantageous when training data are scarce. As a result, whereas CNN-based models can effectively capture the local texture and morphological cues of rice grains, ViT struggles to generalize under the same conditions.

**Figure 10:**
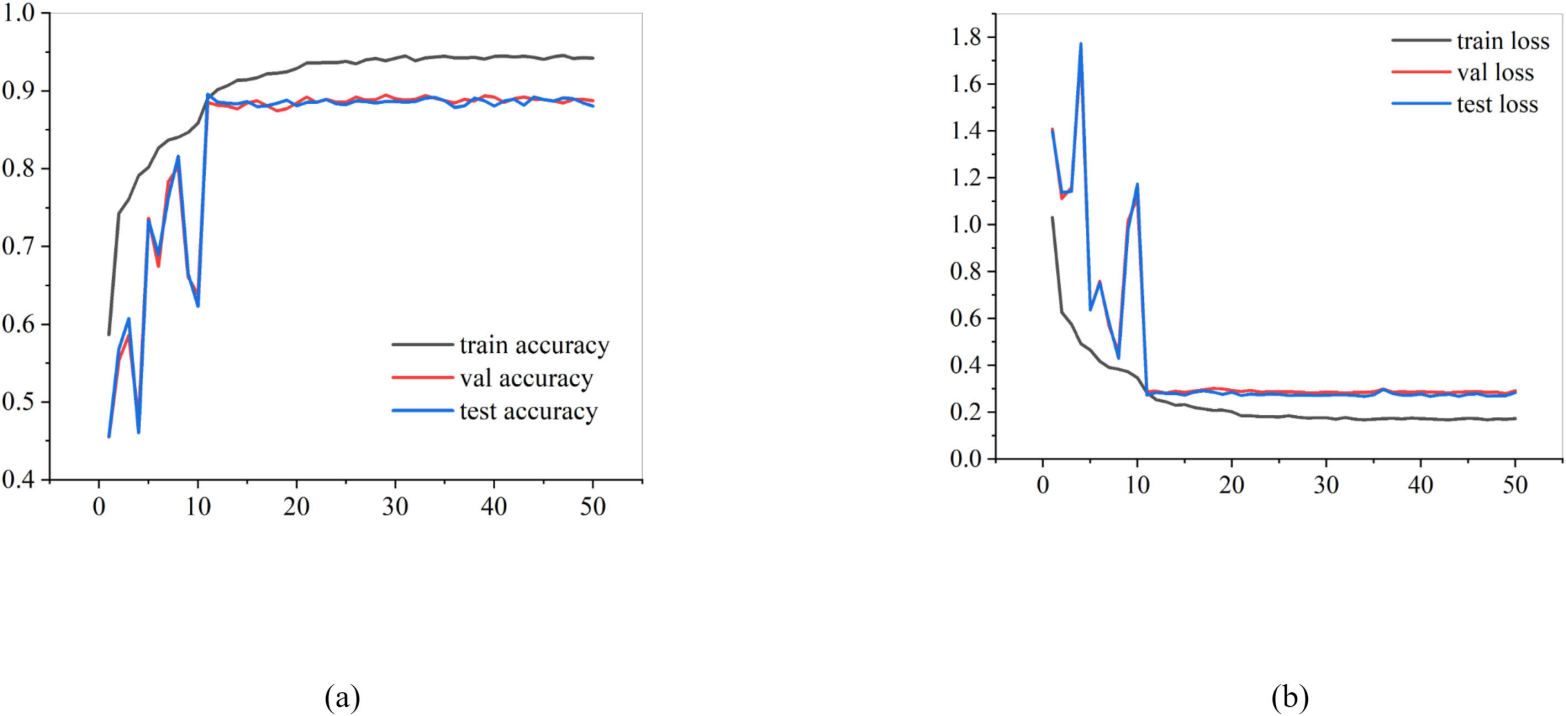
**(a)** Training–val–test accuracy of RiceLCNN for 50 epochs (epoch 0 to 50) **(b)** Training–val–test loss of RiceLCNN for 50 epochs (epoch 0 to 50).

Regarding key performance evaluation metrics, RiceLCNN achieved the highest scores across accuracy (89.78%), recall (89.93%), and F1 score (89.81%), outperforming all other comparison models. It demonstrated superior stability and robustness, particularly in negative class identification and marginally classified sample discrimination. **Figure 10** illustrates the accuracy and loss trends of RiceLCNN during training, validation, and testing. As shown in **Figure 10a**, the model’s accuracy rapidly improves and stabilizes within the first 15 to 20 training epochs. The training set accuracy approaches 95%, while the validation and test set accuracies stabilize around 89% with no noticeable overfitting. The loss curve in **Figure 10b** further validates the model’s convergence and training efficiency, with training, validation, and testing losses all rapidly decreasing and converging below 0.2 within 20 epochs.

**Figure 11:**
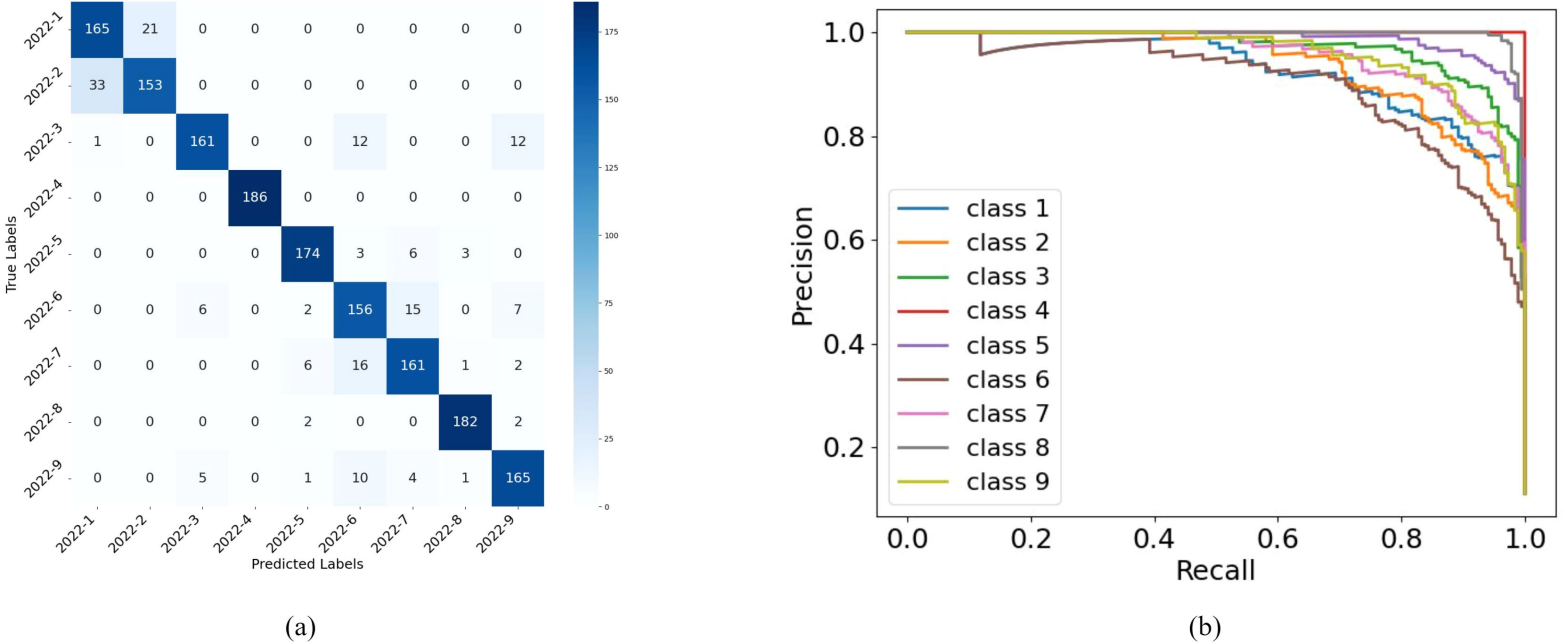
**(a)** Confusion matrix **(b)** precision recall curve of RiceLCNN model on test set of rice grain dataset.

**Figure 11** visualizes RiceLCNN’s classification performance on the test set. **Figure 11a** shows the confusion matrix for nine rice grain categories. The diagonal region indicates high recognition accuracy across all categories with minimal classification error. **Figure 11b** displays the precision-recall (PR) curves for each category. The PR curves for most categories approach the upper-right corner, indicating that the model maintains high precision while preserving high recall, demonstrating strong overall classification robustness.

#### 4.4.3 Public dataset evaluation and generalization ability verification

**Figure 12:**
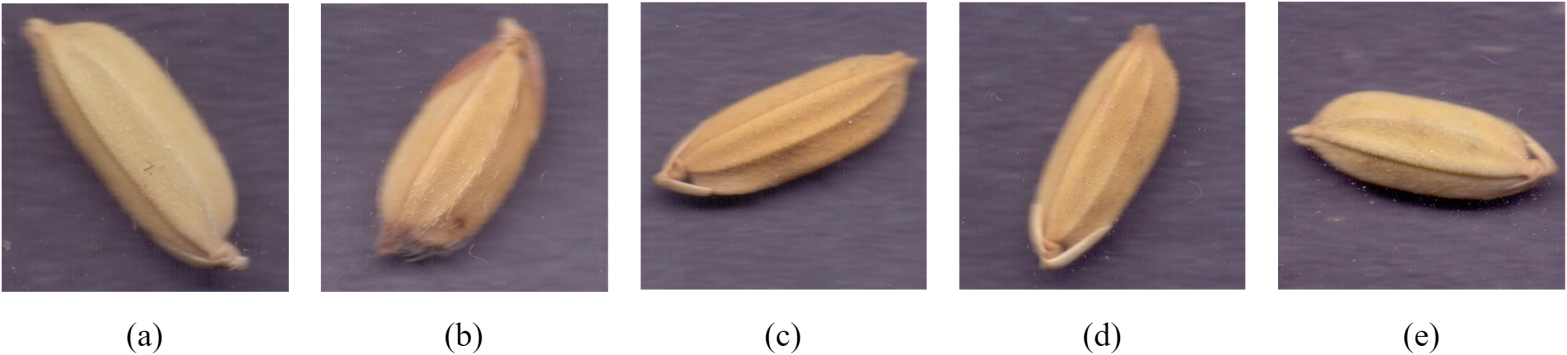
**(a)** Rice variety: Jehlum; **(b)** Rice variety: Mushkibudji; **(c)** Rice variety: Sr-1; **(d)** Rice variety: Sr-2; **(e)** Rice variety: Sr-3.

To further verify the generalization ability and classification robustness of the RiceLCNN model in different data domains, this paper selected the public rice seed image dataset provided by Srinagar, Sher-Kashmir Agricultural and Technological University (SKUAST) as an independent testing platform (Mohi Ud Din et al., 2024). The dataset includes five typical rice varieties: Jehlum, Mushkibudji, Sr-1, Sr-2, and Sr-4, comprising a total of 4,748 high-quality images (as shown in **Figure 12**). The dataset was collected using a high-resolution flatbed scanner (HP Scanjet 200), set to 200 dpi optical resolution and 24-bit color depth. Each image contains 24 seeds of the same rice variety, arranged randomly, with the background covered by black paper to ensure imaging consistency. All images are saved in PNG format, maximizing the preservation of grain arrangement, detailed features, and appearance differences, and presenting a certain level of image complexity and challenge.

**Figure 13:**
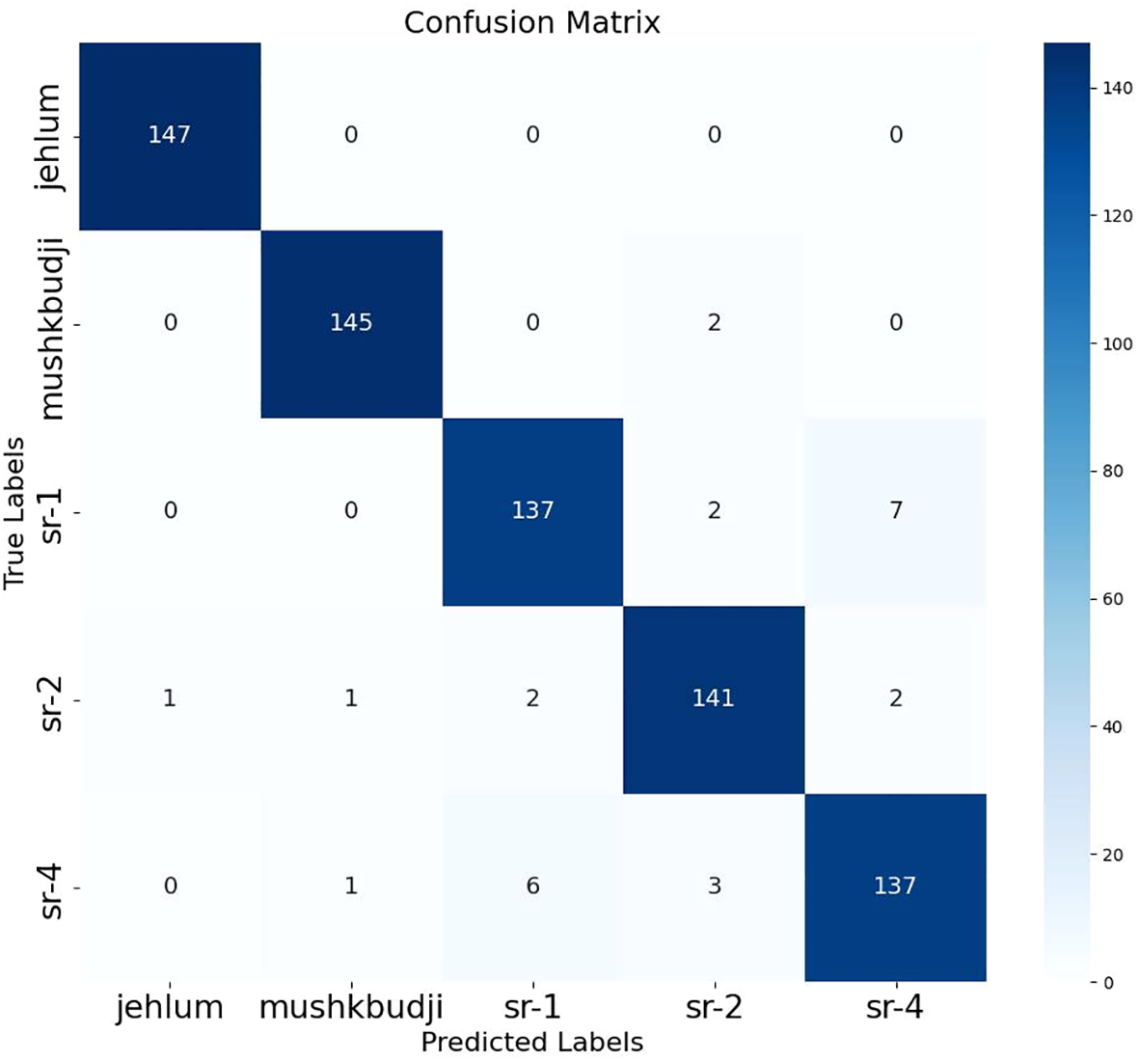
Confusion matrix of RiceLCNN on the public dataset.

For training and evaluation, the dataset was divided into a training set (3,278 images), a validation set (735 images), and a test set (735 images). The test set was used for independent evaluation of the RiceLCNN model, with its classification performance shown in **Table 7** and the confusion matrix shown in **Figure 13**. The results show that RiceLCNN achieves excellent performance on this public rice dataset, with recognition accuracy and recall rates for the Jehlum and Mushkibudji varieties approaching or reaching 100%, and F1 scores of 99.66% and 98.64%, respectively. The F1 scores for the remaining three classes, Sr-1, Sr-2, and Sr-4, are 94.16%, 95.59%, and 93.52%, respectively. The overall accuracy of the model is 96.32%, and the Macro average metric remains between 96.31% and 96.32%, demonstrating good generalization ability and stability.

**Table 7:** Evaluation report of RiceLCNN on public datasets.

Rice variety	P (%)	R (%)	F1 (%)
Jehlum	99.32	100.00	99.66
Mushkibudji	98.64	98.64	98.64
Sr-1	94.48	93.84	94.16
Sr-2	95.27	95.92	95.59
Sr-3	93.84	93.20	93.52
Accuracy (%)			96.32
Macro avg (%)	96.31	96.32	96.32

As shown in **Figure 13**, RiceLCNN achieved 100% accurate classification for Jehlum and Mushkibudji samples; There is some confusion between Sr series varieties, primarily between Sr-1 and Sr-2, as well as Sr-4, but the overall misclassification rate is low, indicating that the model has high discriminative ability in most categories and maintains good robustness in categories with similar phenotypic characteristics.

### 4.5 Implementation and deployment

**Figure 14:**
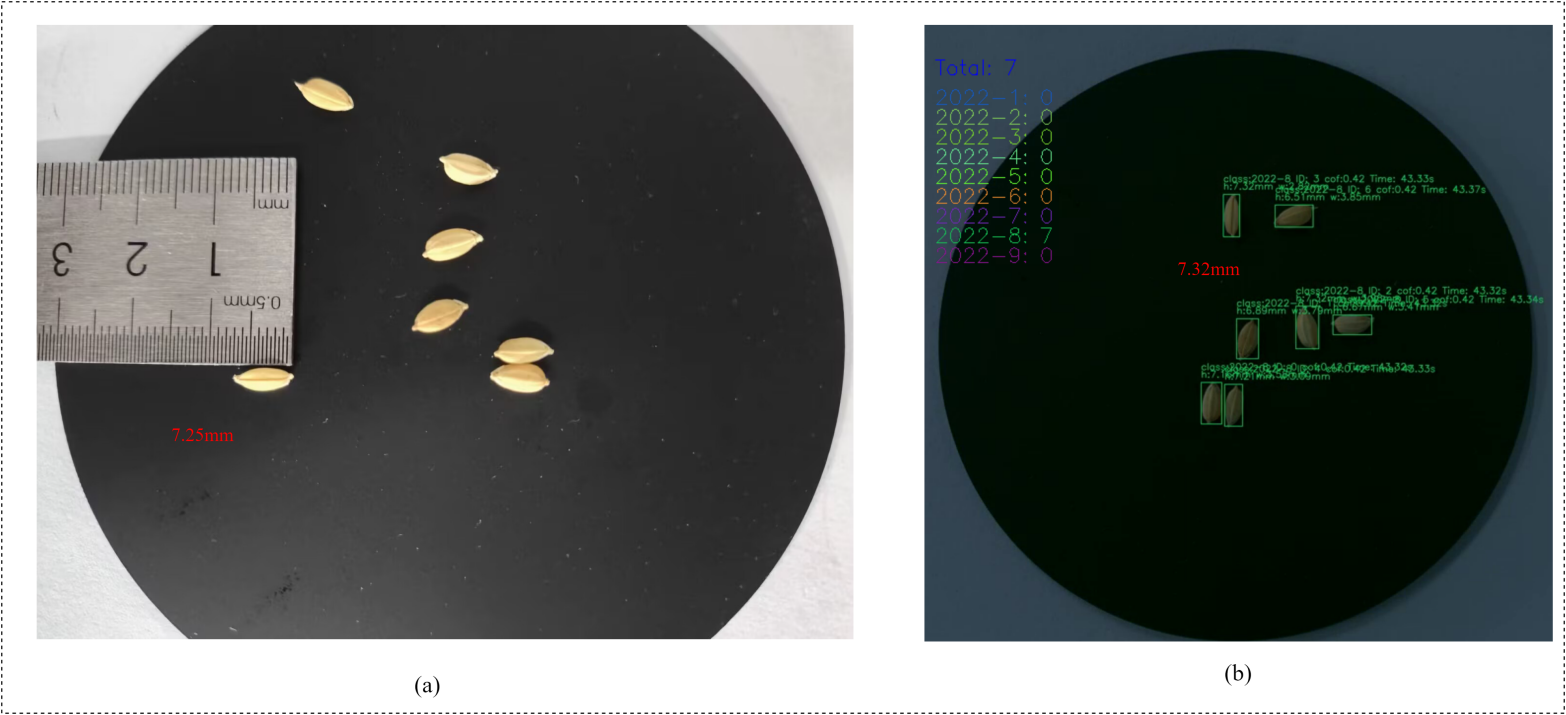
**(a)** Measure the actual length of one of the rice seeds; **(b)** ”Total” indicates that a total of 7 rice grains were identified, all belonging to the variety 2022-8 (actually also for 2022-8). For each individual grain, “class” denotes the predicted category, “ID” represents the unique identity assigned during tracking, and “Time” indicates the tracking duration. “h” and “w” refer to the grain’s length and width in millimeters, respectively. “cof” refers to the classification confidence score—the higher the value, the more reliable the classification result (note: this is the confidence of the classification, not of the detection).

To balance mobile inference efficiency and recognition accuracy, this paper constructs an integrated rice seed recognition system incorporating four key components: “lightweight detection—fine-grained classification—online tracking—morphometric measurement.” YOLOv11-LA handles instance-level localization, RiceLCNN performs single-grain classification, DeepSORT maintains cross-frame ID consistency, and the sub-pixel measurement module outputs phenotypic metrics like length and width (**Figure 14**). Experimental results demonstrate prediction-to-actual measurement errors within 0.1 mm.

The system is deployed on a Jetson Orin Nano 8 GB platform (single input channel, batch = 1) with *Super* power mode enabled; key hardware parameters are listed in **Table 8**. This platform employs a Unified Memory Architecture (UMA), where the GPU lacks dedicated VRAM and shares system memory with LPDDR5.

**Table 8:** Key specifications of Jetson Orin Nano 8GB (by power mode).

INT8 performance (TOPS)	Memory capacity	Memory bandwidth (GB/s)	GPU memory
67	8GB LPDDR5	102	Unified memory

To evaluate the performance of different “detection/classification” combinations, we employ a unified pipeline: “detection → tracking → classification → measurement.” To ensure comparability, all combinations use the same input, with consistent threshold settings and post-processing strategies across configurations. Reported metrics include: throughput FPS (steady-state mean ± standard deviation), end-to-end latency percentiles p50–p99, GPU memory peak, and process resident memory (RSS) peak. All tests were conducted after 5 warm-up passes, with statistics aggregated from 100 consecutive inferences. Deployment results for both input resolutions are shown in **Table 9**.

**Table 9:** Jetson Orin 8GB (batch=1) deployment metrics.

Pipeline	Input(px)	FPS	p50–p99(ms)	GPU mem(GB)	RSS(GB)
YOLOv11-LA–RiceLCNN	1080×1080	14 ± 1	71–85	0.1	1.3
YOLOv11n–Xception	1080×1080	12 ± 1	80–95	0.2	1.4
YOLOv11-LA–RiceLCNN	3200×3200	4 ± 1	209–227	0.6	1.3
YOLOv11n–Xception	3200×3200	2 ± 1	333–362	1.9	1.4

Furthermore, to characterize the speed-accuracy tradeoff, we selected YOLOv11n as a control detector alongside the YOLOv11–LA + RiceLCNN baseline. For the control classifier, we employed Xception, the second-highest performing model on this dataset after RiceLCNN combined as YOLOv11n–Xception. Both approaches were evaluated under identical post-processing and evaluation protocols to highlight the impact of model size and computational load on real-time performance.

## 5 Discussion

Experimental results indicate differences in classification accuracy among fertilization treatments: First, 2022–1 and 2022–2 exhibited the most pronounced bidirectional misclassification, suggesting that grain visible phenotypes remain highly similar when both “straw” and “enzymes” factors vary simultaneously.

Second, 2022–6 and 2022–7 exhibited misclassification almost exclusively between themselves, differing solely in the application of enzymes. This suggests enzymes exert marginal effects on grain appearance (primarily image-visible phenotype and color/chalkiness phenotypes), as illustrated in 11. This aligns with the established understanding that “nitrogen fertilizer primarily influences taste-related physicochemical properties, exerting limited and dose-dependent effects on appearance phenotypes” (Guo et al., 2023; Liang et al., 2021; Shi et al., 2022). In contrast, the classification of 2022–4 and 2022–8 was nearly “pure”, both receiving combined chemical fertilizer application. This suggests chemical fertilizer treatment generates stable phenotypic signals readily captured by visual models, aligning with field evidence that “optimized nitrogen-potassium management significantly reduces chalkiness incidence and severity” (Guo et al., 2024; Zhang et al., 2025).

Building upon this, we deployed a “detection-tracking-classification-quantification” pipeline on a Jetson Orin 8GB edge device: YOLOv11-LA serves as the front-end detector, integrated with DeepSORT for continuous instance-level ID tracking. RiceLCNN then performs fine-grained single-grain classification, coupled with sub-pixel measurement (0.1mm error) to obtain key phenotypic traits including length, width, aspect ratio, and roundness. At 1080×1080 input resolution, the lightweight YOLOv11-LA-RiceLCNN combination achieves a 16.7% FPS improvement over YOLOv11n–Xception with reduced latency. With p50/p99 latency decreasing by approximately 11.3% and 10.5%, respectively. GPU memory and process memory usage were reduced by 50% and 7.1%, respectively. At the high-resolution input of 3200×3200, the lightweight combination’s advantages became even more pronounced: FPS increased from 2 to 4, p50/p99 latency decreased by approximately 37.2% and 37.3% respectively, GPU memory dropped from 1.9GB to 0.6GB (a 68.4% reduction), and RSS memory decreased by 7.1%. As shown in **Table 9**, this demonstrates that the lightweight combination excels in high-resolution scenarios while meeting edge device online processing constraints. In rice variety identification, higher resolutions provide finer-grained texture and contour information. Furthermore, measured frame rates and resource utilization demonstrate the system’s online processing capability. It supports rapid screening and parent selection for breeding and high-throughput phenotyping, random sampling and traceability for variety admixing, and quantitative assessment of treatment effects (e.g., fertilization, crop residue incorporation, enzyme application) within the field-to-postprocessing workflow. On quality control and processing lines, thresholding rules based on length, area, roundness, and variety category can generate actionable grade labels and anomaly alerts. Leveraging DeepSORT significantly reduces duplicate counting risks, enabling flexible switching between sampling and offline verification. Furthermore, research indicates that cross-varietal recognition rates generally exceed those of intra-varietal fertilization treatments, as shown in **Table 7**. This suggests that the explanatory power of genotype main effects surpasses that of fertilizer efficacy main effects, aligning with findings that rice quality is jointly regulated by genotype-environment interactions, with genotype contributions often dominant (Yu et al., 2023; Chen et al., 2012).

It should be noted that this study primarily covers imaging scenarios with “adhesion but minimal occlusion”; under conditions of severe overlap or strong occlusion, front-end detection may require integration with instance segmentation or geometric separation to maintain recall. Domain shifts across camera positions and lighting conditions may also cause performance fluctuations, necessitating mitigation through small-scale retraining or adaptive strategies. Concurrently, potential confounders such as soil moisture, pest/disease pressure, microclimate, and variations in harvest and imaging batches may still influence phenotypic expression and model classification.

## 6 Conclusions and future work

This study proposes and validates an integrated intelligent rice seed recognition system encompassing detection, tracking, classification, and morphometric analysis, achieving a favorable balance between model lightweighting, recognition accuracy, and edge-deployability. It employs YOLOv11-LA as the front-end detector, utilizes DeepSORT for instance-level ID association, and leverages RiceLCNN for precise single-grain classification. Combined with sub-pixel measurement, it outputs key traits including length, width, aspect ratio, and roundness. The system operates stably on edge devices like Jetson Orin 8 and GB, enabling real-time processing for breeding screening, data collection, and quality monitoring in processing lines. A systematic analysis based on confusion matrices further indicates that, compared to fertilization treatments, variety (genotype) explains a higher proportion of seed appearance phenotypes. Among these, enzyme application alone has a marginal impact on visible phenotypes, while differences induced by chemical fertilizer treatments are more readily captured by visual models. These findings provide an efficient, stable, and engineering-feasible technical pathway for intelligent seed recognition and crop phenotyping.

Future work will focus on enhancing the system’s extrapolation capability and application depth through the following directions: (1) Incorporate multi-modal sensing (e.g., near-infrared/hyperspectral imaging) and cross-modal feature alignment to enhance recognition and quantitative characterization capabilities across diverse physiological states and environmental conditions; (2) Conduct robustness enhancement and domain adaptation studies under non-ideal natural conditions (backlighting, mud spots, strong shadows, cross-camera/cross-lighting), ensuring model robustness and interpretability through hierarchical cross-validation and uncertainty estimation; (3) Promote deep integration with field agricultural machinery and IoT nodes to construct a closed-loop “perception-decision-execution” control prototype. (4) Extend testing to multi-varietal datasets and real field environments, and explore integration with breeding programs to support large-scale phenotyping and accelerate genetic improvement pipelines. This supports online grading, anomaly removal, and adaptive adjustment of operational parameters, further advancing the digitalization and precision of agricultural production.

## Data Availability

The datasets presented in this study can be found in online repositories. The names of the repository/repositories and accession number(s) can be found below: https://github.com/5120191452/RiceLCNN.
